# Mechanically Tunable Hydrogels with Self-Healing and Shape Memory Capabilities from Thermo-Responsive Amino Acid-Derived Vinyl Polymers

**DOI:** 10.3390/gels9100829

**Published:** 2023-10-19

**Authors:** Shin-nosuke Nishimura, Dan Sato, Tomoyuki Koga

**Affiliations:** Department of Molecular Chemistry and Biochemistry, Faculty of Science and Engineering, Doshisha University, Kyotanabe 610-0321, Kyoto, Japan; ctwj0749@mail4.doshisha.ac.jp

**Keywords:** amino acid-derived vinyl polymer, hydrogel, post-crosslinking, thermo-responsiveness, shape memory, self-healing, moldability, mechanical tunability

## Abstract

In this study, we report the fabrication and characterization of self-healing and shape-memorable hydrogels, the mechanical properties of which can be tuned via post-polymerization crosslinking. These hydrogels were constructed from a thermo-responsive poly(*N-*acryloyl glycinamide) (NAGAm) copolymer containing *N*-acryloyl serine methyl ester (NASMe) units (5 mol%) that were readily synthesized via conventional radical copolymerization. This transparent and free-standing hydrogel is produced via multiple hydrogen bonds between PNAGAm chains by simply dissolving the polymer in water at a high temperature (~90 °C) and then cooling it. This hydrogel exhibited moldability and self-healing properties. The post-polymerization crosslinking of the amino acid-derived vinyl copolymer network with glutaraldehyde, which acts as a crosslinker between the hydroxy groups of the NASMe units, tuned mechanical properties such as viscoelasticity and tensile strength. The optimal crosslinker concentration efficiently improved the viscoelasticity. Moreover, these hydrogels exhibited shape fixation (~60%)/memory (~100%) behavior owing to the reversible thermo-responsiveness (upper critical solution temperature-type) of the PNAGAm units. Our multifunctional hydrogel, with moldable, self-healing, mechanical tunability via post-polymerization crosslinking, and shape-memorable properties, has considerable potential for applications in engineering and biomedical materials.

## 1. Introduction

In recent years, considerable attention has been paid to the design of functional hydrogels with a wide range of applications in the biomedical, cosmetic, and industrial fields. Hydrogel materials are useful in various contexts because they possess excellent biocompatibility due to their highly water-swollen network, structural flexibility, and ability to support various substances [1,2]. In addition to these intrinsic properties, hydrogel materials can impart specific functions such as stimuli-responsiveness [3,4], high mechanical toughness [5,6,7], self-healing [8,9,10,11], injectability [12,13,14], and shape fixation/memory [15,16,17] capabilities. Moreover, it has recently become possible to design unique smart gels that combine multiple functions into a single hydrogel [18,19]. These soft materials have been used as scaffolds in tissue engineering, controlled and sustained drug release, wound healing, sensors, actuators, and water absorbents. To fabricate such a smart gel system, designing the crosslinking points and polymer main chains that form the network skeleton is important. Both covalent and noncovalent bonds can be employed as crosslinking points. The former is suitable for improving mechanical strength, whereas the latter is convenient for designing dynamic and reversible structural properties, which are intrinsically required for constructing an external stimuli-responsive product.

Shape-memory hydrogels are fascinating stimuli-responsive hydrogels that can fix temporary shapes and recover their original shape through the reversible and partial formation/deformation of crosslinks in response to external cues (e.g., heat, light, and pH). To date, various reversible noncovalent interactions such as hydrogen bonding [20,21,22,23,24], hydrophobic interactions [25,26], metal–ligand interactions [27,28], and host–guest interactions [29,30] have been used to achieve shape-memory behavior. Introducing these reversible interactions into a hydrogel system is necessary to fix the network structure temporarily. However, this often results in a gel with weak mechanical strength. To overcome this trade-off, we previously prepared chemically crosslinked poly(*N*-acryloyl glycinamide) (PNAGAm) hydrogels [24]. PNAGAm is a biocompatible amino acid-based vinyl polymer with two amide bonds in its units, namely, two pairs of H-donors (NH) and H-acceptors (C=O), providing strong and reversible hydrogen bonds between the polymer chains [31,32,33]. Owing to its unique molecular structure, PNAGAm exhibits upper critical solution temperature (UCST) behavior in water at low concentrations (< 2 wt %) and forms hydrogels showing thermo-reversible gel–sol transition at higher concentrations. Our previous study demonstrated that a chemically crosslinked PNAGAm hydrogel successfully provided both good mechanical strength and shape fixation/memory functions, based on two distinct physical (thermo-sensitive) and chemical (thermo-stable) cross-linkages [24]. However, in this system, the initial shape and physical properties of the hydrogel are determined during polymerization. Therefore, modifying them after polymerization is difficult. Thus, these hydrogels inherently lack dynamic properties such as moldability and mechanical tunability, which are important in practical use. For example, the elastic modulus of hydrogels influences cell behavior, including adhesion, proliferation, and differentiation, when used as a scaffold for tissue engineering [12,34,35]. In addition, it is a useful factor for controlling the sustained release of inclusions because the elastic moduli of hydrogels are closely related to their crosslinking density [14,36]. Therefore, fabricating a new hydrogel system that can freely mold a shape and tune its mechanical properties while maintaining shape fixation/memory functions is an important challenge in developing innovative three-dimensional (3D) matrices for versatile application potential.

**Figure 1:**
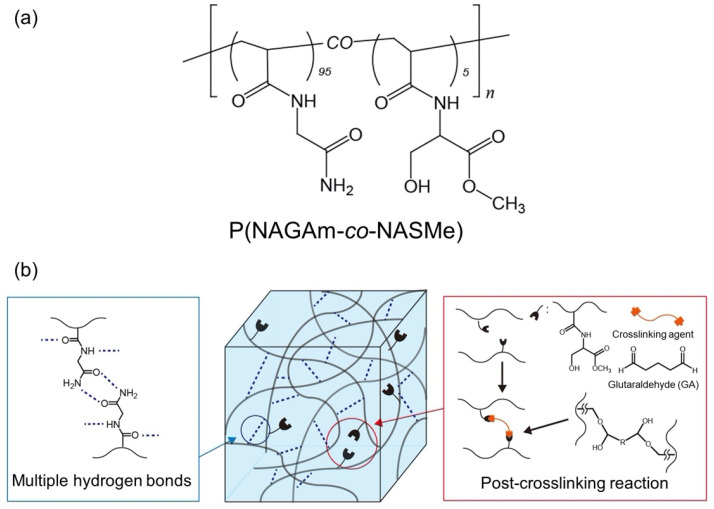
(**a**) Chemical structure of the P(NAGAm-*co*-NASMe) used in this study. (**b**) Schematic illustration of the post-crosslinking of P(NAGAm-*co*-NASMe) hydrogel with glutaraldehyde (GA).

In this study, we report a novel amino-acid-derived vinyl copolymer, PNAGAm-containing *N*-acryloyl serine methyl ester (NASMe), as a supramolecular hydrogel material with reactive functional groups. A moldable hydrogel was formed from P(NAGAm-*co*-NASMe) via simple thermal treatment (heating/cooling) in water. In this system, PNAGAm-based hydrogels were crosslinked with glutaraldehyde (GA) after gelation to tune their mechanical properties (Figure 1). The hydrogel properties, including thermo-responsive, mechanical, shape fixation/memory, and self-healing behaviors, were comprehensively investigated with respect to the influence of post-polymerization crosslinking (post-crosslinking). Integrating multiple smart functions into a single hydrogel can meet the requirements for various industrial, biomedical, and cosmetic applications.

## 2. Results and Discussion

### 2.1. Preparation of Hydrogels from Amino Acid-Derived Vinyl Polymer

Thermo-sensitive polymers are useful building blocks for designing stimuli-responsive hydrogels with dynamic and reversible physical cross-linkage. In these systems, polymer–polymer and polymer–water interactions vary reversibly through a change in the conformation and/or hydration state of the constituent polymer in response to the environmental temperature. To date, a wide variety of thermo-responsive polymers have been successfully developed for both the lower critical solution temperature (LCST) and UCST types [37]. Among these, amino acid-derived vinyl polymers are among the most fascinating thermo-responsive and bio-based polymers [38,39,40,41,42,43,44,45]. Their response type (LCST or UCST) and temperature can be controlled by varying the type of amino acid (20 types) and terminal structures [40,43,44]. They are also easy to synthesize and are biocompatible [32,33,41]. We previously constructed an amino acid-derived vinyl monomer library from various amino acids (Gly, Ala, β-Ala, Val, Leu, Phe, Ser, and Lys) and prepared diverse LCST/UCST polymers by selecting and/or combining the monomers from the library [45].

In this study, we focused on UCST-type PNAGAm copolymer as a network frame for shape-memory hydrogels. As described above, the chemically crosslinked PNAGAm hydrogels exhibit good shape fixation/memory function but have less moldability and tunable physical properties once the gel is formed. To address this issue, PNAGAm-containing Ser-based reactive hydroxy groups, which allow post-crosslinking of the gel, were synthesized via the simple copolymerization of *N*-acryloyl glycinamide (NAGAm) and NASMe. Post-crosslinking of hydrogel materials is a useful strategy to tune their mechanical properties, particularly in biopolymer-based self-assembling systems [46,47].

Copolymerization of NAGAm with NASMe (5 mol%) was performed in water/DMSO (*v*/*v* = 11/4) using ammonium persulfate (APS) as an initiator. A small amount of polyethylene glycol diacrylate (PEGDA; 0.05 mol% of the total amino acid monomers) was added to increase the molecular weight. The target P(NAGAm-*co*-NASMe) copolymer was successfully obtained as a white powder after purification via dialysis and lyophilization. The chemical structures were characterized using ^1^H-NMR spectroscopy (Figures S1–S3 in the Supplementary Materials).

**Figure 2:**
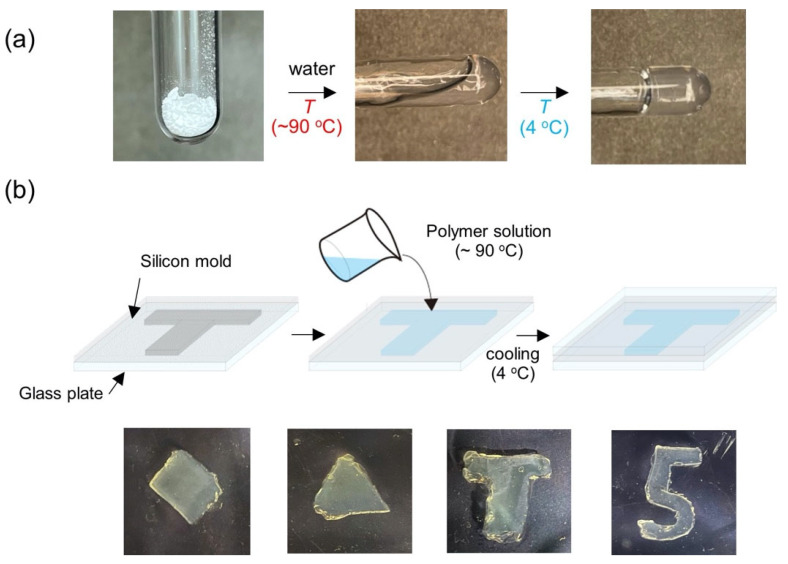
(**a**) Photographs of P(NAGAm-*co*-NASMe) during hydrogel formation by thermal treatment. (**b**) Molding protocol of P(NAGAm-*co*-NASMe) hydrogel (top) and photographs of different hydrogel shapes (bottom). The hydrogel can be freely molded by pouring the polymer solution (~90 °C) into differently shaped molds and cooling.

At low concentration (1 wt %), the P(NAGAm-*co*-NASMe) exhibited reversible UCST behavior in water, with a gradual transition curve of between 4 and 50 °C, even with the NASMe units (Figure S4), as was observed in the PNAGAm homopolymer [31,33]. In a concentrated condition (4 wt %), the polymer formed a transparent and self-supporting hydrogel when dissolving the polymer powder in water at a high temperature (~90 °C) using a microwave oven and then cooling it (Figure 2a). This is due to the multiple thermally reversible hydrogen bonds among the NAGAm units. Using this thermal response, this hydrogel can be molded into any desired shape by pouring the polymer solution into molds of different shapes, such as rectangular, triangular, T-, and number 5-shapes (Figure 2b). Thus, the initial shape of the hydrogel can be easily and freely changed without the need for special processing of the hydrogel material. Notably, the hydrogel was remoldable into different shapes via heating (90 °C)/cooling treatment, although the overall shape was maintained up to 70 °C. These gel properties are convenient for practical applications such as wound healing and 3D bioprinting materials [48,49].

### 2.2. Hydrogel Property of P(NAGAm-co-NASMe) Post-Crosslinked with GA

**Figure 3:**
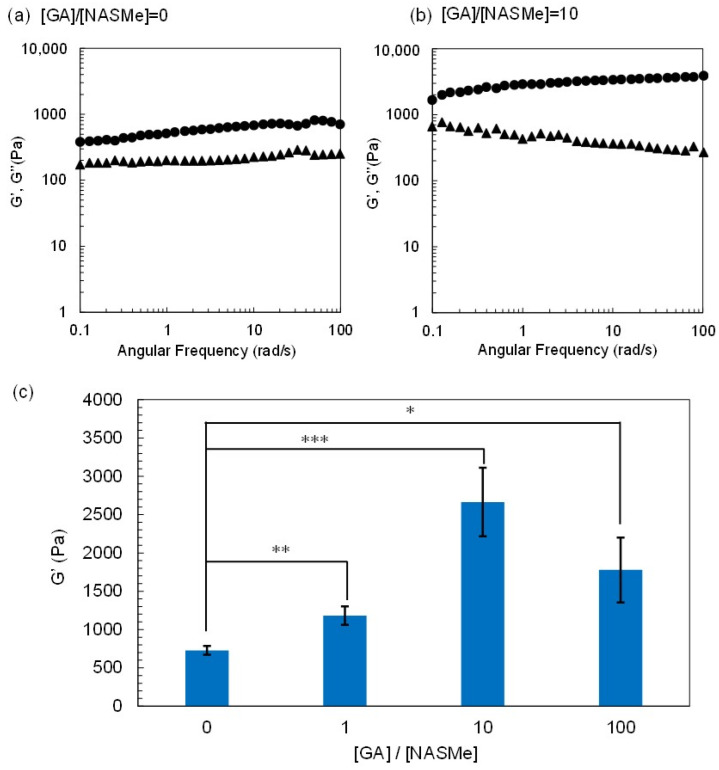
(**a**,**b**) Rheological analysis of P(NAGAm-*co*-NASMe) hydrogel before ([GA]/[NASMe] = 0) (**a**) and after post-crosslinking by GA ([GA]/[NASMe] = 10 (10 equiv.)) (**b**). The storage moduli (G′) (closed circle) and loss moduli (G″) (closed triangle) are plotted as a function of frequency. (**c**) Comparison of G′ values (at 6.3 rad/s, 1% strain) for various P(NAGAm-*co*-NASMe) hydrogels obtained after the post-crosslinking reaction under different GA concentrations. The error bars represent the mean standard deviation (*N* = 3). * *p* < 0.05, ** *p* < 0.01, *** *p* < 0.005.

**Figure 4:**
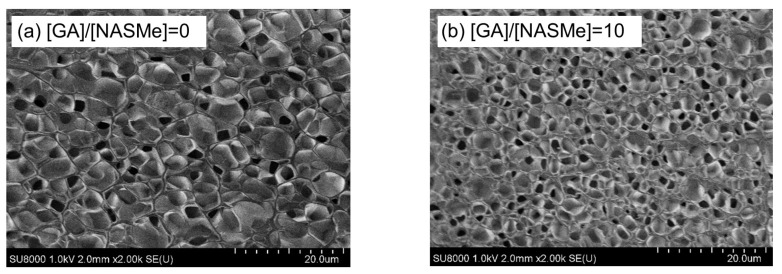
SEM images of P(NAGAm-*co*-NASMe) hydrogels before ([GA]/[NASMe] = 0) (**a**) and after post-crosslinking by GA ([GA]/[NASMe] = 10) (**b**). The hydrogels were flash-frozen in liquid nitrogen from a standing temperature of 4 °C and immediately freeze-dried.

Post-crosslinking of the P(NAGAm-*co*-NASMe) hydrogel with GA was performed by immersing the prepared hydrogel (4 °C) in GA aqueous solutions for 1 h at 4 °C. To verify how the GA concentration affects the post-crosslinking reactions and resultant hydrogel properties, different ratios of GA concentration (crosslinker) to NASMe units ([GA]/[NASMe]) were tested, ranging from 0 to 100. Figure 3a,b shows the dynamic viscoelasticity of the hydrogels after the post-crosslinking reaction ([GA]/[NASMe] = 0 and 10). Both hydrogels, even for non-post-crosslinked gel ([GA]/[NASMe] = 0), demonstrated that the storage modulus (G′) was nearly constant over the entire frequency (0.1–100 rad/s) and was significantly larger than the loss modulus (G″). This indicates the long relaxation time of the network and a stable gel structure. However, the G′ value of the hydrogel after post-crosslinking increased ~3.7 times compared to the non-crosslinked gel, suggesting the successful formation of chemical cross-linkages between aldehyde groups of GA and hydroxy groups on the NASMe units, which enhances the crosslinking density. In fact, significant differences were observed in the surface morphologies of the hydrogels (Figure 4). SEM images of the freeze-dried hydrogels from a standing temperature of 4 °C demonstrated that both gels possessed similar porous structures, but the average pore size of the post-crosslinked gel (1.67 μm) was significantly smaller than that of the non-crosslinked gel (3.63 μm), resulting in the denser porous structure of the post-crosslinked gel. Figure 3c shows the post-crosslinking efficiency of the P(NAGAm-*co*-NASMe) hydrogel at different GA concentrations. The gel stiffness (G′) increased with increasing GA concentration, from ~750 Pa ([GA]/[NASMe] = 0) to 2800 Pa ([GA]/[NASMe] = 10), owing to post-crosslinking under this experimental condition. However, when the GA concentration was too high ([GA]/[NASMe] = 100), the gel stiffness decreased. This may be attributed to an increase in the proportion of the GA that reacts with only one hydroxyl group. In addition, the possibility of long-range cross-linkage with long GA chains cannot be excluded, since GA is known to self-polymerize into oligomer/polymer at high concentrations [50]. Thus, GA concentration is an important factor for achieving an efficient post-crosslinking reaction in the P(NAGAm-*co*-NASMe) hydrogel.

**Figure 5:**
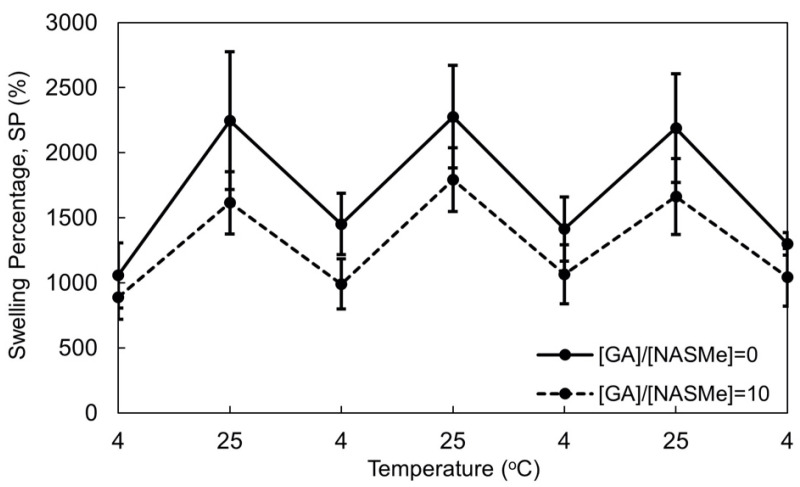
Reversible changes between the swelling (25 °C) and deswelling (4 °C) of P(NAGAm-*co*-NASMe) hydrogels with ([GA]/[NASMe] = 10) (dashed line) and without ([GA]/[NASMe] = 0) (solid line) post-crosslinking. The error bars represent mean standard deviation (*N* = 4).

Subsequently, we investigated the effect of post-crosslinking on the temperature-responsive swelling behavior of the hydrogel. Figure 5 compares the reversible change in swelling and shrinking behavior of the hydrogels with and without post-crosslinking when the hydrogels were alternately immersed in water at 4 and 25 °C. The changes in the swelling percentages were completely thermo-reversible in both cases. These thermal behaviors can be attributed to the partial breakdown and reformation of hydrogen bonds between the NAGAm units. Notably, the post-crosslinking reaction did not affect the intrinsic temperature response of the hydrogel; however, the absolute swelling ratio was smaller than that of non-post-crosslinked gel at both 4 and 25 °C, owing to the formation of chemical cross-linkages.

### 2.3. Shape-Memory and Self-Healing Behaviors of P(NAGAm-co-NASMe) Hydrogels

**Figure 6:**
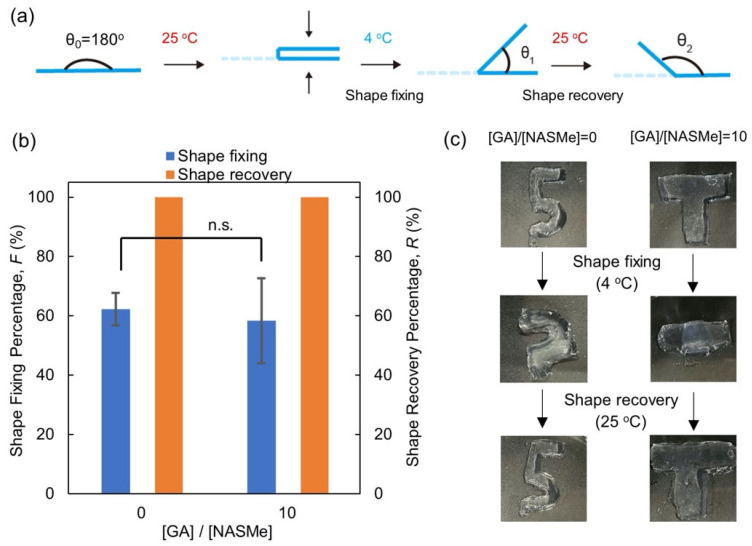
(**a**) Schematic of the shape memory experiment. (**b**) Shape fixing and recovery percentages of P(NAGAm-*co*-NASMe) hydrogels with ([GA]/[NASMe] = 10) and without ([GA]/[NASMe] = 0) post-crosslinking. The error bars represent the mean standard deviation (*N* = 5). n.s.: not significant. (**c**) Photographs of the shape memory behavior of molded P(NAGAm-*co*-NASMe) hydrogels with complex shapes.

Based on thermo-reversible physical cross-linkage, these hydrogels exhibit shape fixation and memory functions in response to temperature manipulation. Figure 6 shows the shape-memory behaviors of the P(NAGAm-*co*-NASMe) hydrogels with ([GA]/[NASMe] = 10 (10 equiv.)) and without post-crosslinking ([GA]/[NASMe] = 0 (0 equiv.)). The shape-fixing and recovery percentages were quantitatively evaluated using straight-shaped hydrogels. In brief, straight-shaped gels (θ_0_ = 180°) were first folded in half by applying external force at 25 °C and were then fixed at 4 °C. Then, the temporarily shaped hydrogels were reheated at 25 °C. The shape-fixing percentage (*F* (%)) and shape-recovery percentage (*R* (%)) were evaluated from the bending angles of the temporary (θ_1_) and recovered (θ_2_) shapes (Figure 6a). All hydrogels showed relatively good shape fixation (*F* = 62% (without post-crosslinking) and 58% (with post-crosslinking)) and excellent shape recovery (*R* = 100%) (Figure 6b). This is because the multiple hydrogen bonds among the NAGAm units, which are partially deformed/reformed by temperature changes without losing the overall gel shape, temporarily lock the structure in place by rearranging the hydrogen bonds to fit the deformed network structures. Thus, this hydrogel system exhibited shape memory behavior even when the post-crosslinking reaction occurred in the NASMe units. Therefore, the NAGAm and NASMe units do not interfere with each other and function independently. As shown in Figure 6c, shape fixation and recovery can be accomplished, even for molded hydrogels with complex shapes, regardless of the post-crosslinking reaction.

**Figure 7:**
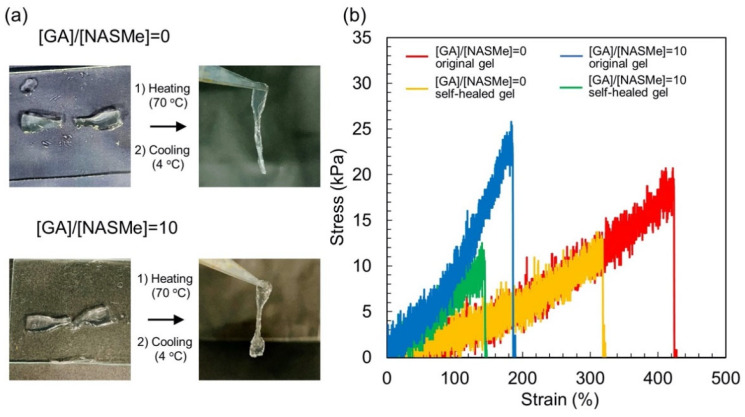
Self-healing behavior of P(NAGAm-*co*-NASMe) hydrogels. (**a**) Photographs of self-healing behavior of P(NAGAm-*co*-NASMe) hydrogels with ([GA]/[NASMe] = 10) and without ([GA]/[NASMe] = 0) post-crosslinking. (**b**) Representative stress–strain curves of original (red and blue) and self-healed (yellow and green) P(NAGAm-*co*-NASMe) hydrogels at 4 °C. P(NAGAm-*co*-NASMe) hydrogels with (blue and green) and without post-crosslinking by GA (red and yellow).

Finally, we investigated the self-healing behavior of the P(NAGAm-*co*-NASMe) hydrogels with and without post-crosslinking by GA. Self-healing is the ability to autonomously restore morphological and mechanical properties after damage and has attracted attention, particularly in the cosmetic and biomedical fields. As shown in Figure 7a, the dumbbell-shaped hydrogels were cut in half using a razor blade. The cut pieces were placed in contact tightly and were then heated (70 °C) and cooled (4 °C). All the hydrogels exhibited self-healing properties that were sufficient to support their weight. Such self-healing properties of these hydrogels are primarily attributed to the reversible hydrogen bonds among the NAGAm units. In fact, when the cutting surfaces of the hydrogels were soaked in a urea solution for 30 min (8 M), a solution that is known to be a hydrogen bond-breaker, no self-healing occurred. Figure 7b shows the representative stress–strain (SS) curves for the original and self-healed hydrogels with and without post-crosslinking. Based on the results of a tensile test of the original hydrogels (Figure 7b, red and blue curves), the mechanical properties of the hydrogels were altered by post-crosslinking, as described in Section 2.2. Post-crosslinking of the hydrogel decreased the elongation at break (from 350–450% to 100–200%) but increased the breaking strength (21 kPa to 26 kPa) and Young’s modulus (3 kPa to 14 kPa). This result is consistent with the trend of the observed elastic modulus change owing to post-crosslinking (Figure 3). Furthermore, the SS curves of the self-healed gels nearly overlapped with the original curves in both cases (Figure 7b; green and yellow curves), indicating the relatively good self-healing abilities of these hydrogels. The gels were cleaved at the cut position with a lower elongation than those of the original gels. The healing efficiency (HE), which was defined in this study as the ratio of the breaking energies of the original and self-healed gels, did not change significantly by post-crosslinking; in the case of HE, the values were 46% without post-crosslinking and 43% with post-crosslinking.

A multifunctional hydrogel system with moldable, mechanically tunable, shape-memorable, and self-healing capabilities was successfully obtained from a thermo-responsive amino acid-derived vinyl polymer.

## 3. Conclusions

In summary, we have successfully designed and prepared an amino acid-based copolymer, P(NAGAm-*co*-NASMe), with reactive serine residues. The hydrogels were formed through multiple hydrogen bonds among the NAGAm units by a simple thermal treatment (heating/cooling) of the polymer in water (4 wt %); thus, they could be molded into any shape. The distinguishing advantages of this hydrogel are its easy synthesis, biocompatible amino acid-based composition, mechanical tunability via post-polymerization crosslinking, and specific multifunctionalities (moldable/self-healable/shape-memorable). The post-crosslinking reaction between Ser residues in the hydrogel with GA at the optimal concentration improves the mechanical properties without losing its self-healing (~45%) and shape fixation (~60%)/memory (~100%) capabilities; for instance, the stiffness (G′) increased ~3.7 times after the reaction. We believe that the amino acid-based smart hydrogel system proposed in this study will be considerably interesting in a wide range of biomedical and cosmetic fields, such as use as a 3D bioscaffold for tissue engineering, sustained release of therapeutics, and 3D bioprinting [32,33,51,52].

## 4. Materials and Methods

### 4.1. Materials and Reagents

Glycinamide hydrochloride and serine methyl ester hydrochloride were purchased from Watanabe Chemical Industry Co, Ltd. (Hiroshima, Japan). Acryloyl chloride, hydrochloric acid (12 M), potassium carbonate, sodium hydroxide, diethyl ether, methanol, ethanol, tetramethylsilane (TMS), ammonium persulfate (APS), sodium trimethylsilyl propane sulfonate (DSS), *N*,*N*-diisopropylethylamine (DIPEA), dichloromethane (DCM), and anhydrous sodium sulfate (Na_2_SO_4_) were purchased from Nacalai Tesque, Inc. (Kyoto, Japan). Deuterium oxide (D_2_O), 25% glutaraldehyde solution, and Wako Gel^®^ FC-40 were purchased from FUJIFILM Wako Pure Chemical, Co. (Osaka, Japan). Deuterated dimethyl sulfoxide (DMSO-*d*_6_) was purchased from Cambridge Isotope Laboratories, Inc. (Tewksbury, MA, USA). Polyethylene glycol diacrylate (PEGDA) was purchased from Tokyo Chemical Industry Co. (Tokyo, Japan) and dialysis membrane (MWCO: 25,000 Da) from Funakoshi Co. (Tokyo, Japan). The glass plates (76 mm × 52 mm) were obtained from Matsunami Glass Industry Co., Ltd. (Osaka, Japan). Glass plates were hydrophobically treated with Siliconize L-25 (Fuji Systems Co., Tokyo, Japan) before use. Silicone rubber plates (1 mm) were obtained from As One Co. (Osaka, Japan). All the reagents were used without further purification.

### 4.2. Measurements

The ^1^H-NMR spectra were measured using a JEOL JNM-ECA500 (JEOL Resonance Co., Ltd., Tokyo, Japan) spectrometer (500 MHz). Transmittance of the polymer solution (1 wt %) was recorded at 700 nm using a JASCO V650ST UV–visible spectrophotometer (JASCO Ltd., Tokyo, Japan). The heating and cooling rate was 0.5 °C/min. The rheological properties of the hydrogels were evaluated using Discover HR-1 (TA Instruments Inc., New Castle, DE, USA) with a Peltier device for temperature control. A solvent trap was used during all measurements to minimize evaporation. The measurement was performed at 4 °C (constant strain: 1%) using a parallel plate (diameter: 20 mm) and the gap was adjusted to approximately 400 μm to ensure that the geometry was filled. Tensile tests of the hydrogels were conducted using a Shimadzu EZ Graph compact tabletop tester (Shimadzu Co., Kyoto, Japan) with a screw-type flat gripper for test fixtures. The hydrogels were prepared using a No. 7 dumbbell-shaped punching blade mold, manufactured by Kobunshi Keiki Co., Ltd. (Kyoto, Japan) (JIS K 6251-7 (ISO37-4)). A tensile speed of 35 mm/min was used for the tensile testing. The cooled hydrogels (4 °C) were used for the tensile tests. The average breaking strength, elongation at break, and Young’s modulus were calculated from the strain–stress curves (*N* = 3). The surface morphology of the hydrogels was evaluated using field-emission scanning electron microscopy (FE-SEM (SU8020), Hitachi High-Technologies Co, Tokyo, Japan). Samples were freeze-dried from 4 °C and attached to a grid. The measurement was performed at an acceleration voltage of 1 kV.

### 4.3. Synthesis of N-Acryloyl Glycinamide (NAGAm) and N-Acryloyl Serine Methyl Ester (NASMe)

*N*-acryloyl glycinamide (NAGAm) and *N*-acryloyl serine methyl ester (NASMe) were synthesized following the method used in our previous paper (detailed procedure, see Supplementary Materials and Scheme S1) [43,45]. The chemical structures were confirmed using ^1^H-NMR spectroscopy (Figures S1 and S2).

^1^H-NMR (NAGAm, DMSO-*d*_6_, TMS): δ 3.72 ppm (2H, –NHC*H*_2_CO–), 5.6 ppm (1H, vinyl (cis), C*H*_2_=CH–), 6.1 ppm (1H, vinyl (trans), C*H*_2_=CH–), 6.3 ppm (1H, vinyl, CH_2_=C*H*–), 7.0–7.5 ppm (2H, –CON*H*_2_), 8.33 ppm (1H, –N*H*CH_2_CO–).

^1^H-NMR (NASMe, DMSO-*d*_6_, TMS): δ 3.65 ppm (3H, –COOC*H*_3_), 3.73 ppm (2H, -C*H*_2_OH), 4.44 ppm (1H, –COC*H*NH–), 5.1 ppm (1H, –CH_2_O*H*), 5.62 ppm (1H, vinyl (cis), C*H*_2_=CH–), 6.1 ppm (1H, vinyl (trans), C*H*_2_=CH–), 6.4 ppm (1H, vinyl, CH_2_=C*H*–), 8.47 ppm (1H, –N*H*CH_2_CO–).

### 4.4. Synthesis of P(NAGAm-co-NASMe)

NAGAm (0.608 g, 4.75 mmol) and NASMe (0.0390 g, 0.250 mmol) were dissolved in a mixture of water/DMSO (11/4 (*v*/*v*)). To this mixed solution, APS (6.47 mg, 0.0283 mmol) and a small amount of polyethylene glycol diacrylate (PEGDA) (0.755 μL, 2.50 μmol) were added as an initiator and a crosslinking agent, respectively (the total volume was 5 mL). The solution was poured into a polymerization tube, followed by three rounds of freeze–vacuum–thaw degasification using liquid nitrogen as a cryogen to remove dissolved oxygen from the reaction solution. Then, the reaction mixture was placed in an oil bath at 70 °C for 18 h (Scheme S1). The in situ ^1^H-NMR analysis confirmed that all the monomers were completely consumed and converted to the polymer under these polymerization conditions. The reaction solution was cooled to ambient temperature and purified via dialysis against distilled water for 3 d, using a dialysis tube with a MWCO of 25,000 Da at ambient temperature. The solvent was removed by lyophilization to obtain the pure polymers as a white solid (P(NAGAm-*co*-NASMe)). The isolated copolymer was relatively insoluble because of its self-assembling nature, and it was only soluble in a highly polar solvent. Therefore, inherent viscosity analysis of the copolymer in 2M NaSCN aqueous solution was conducted at 25 °C, using an Ostwald-type viscometer to evaluate the viscosity-average molecular weight (*M*_v_). The *M*_v_ value of P(NAGAm-*co*-NASMe) was estimated to be approximately *M*_v_ = 1.36 × 10^6^, using the Mark–Houwink–Sakurada equation, as follows: [*η*] = *KM*_v_^α^. Here, the *K* (1.16 × 10^−3^) and α (0.52) values of pure PNAGAm [53] were used to calculate *M*_v_. The chemical structure of the purified polymer was characterized using ^1^H-NMR spectroscopy (Figure S3).

^1^H-NMR (D_2_O, DSS, *n* means polymerization degree): δ 0.8–3.0 ppm (2H × 0.95*n*, methylene of main chain for NAGAm units; 1H × 0.95*n*, methine of main chain for NAGAm units; 2H × 0.05*n*, methylene of main chain for NASMe units; 1H × 0.05*n*, methylene of main chain for NASMe units), 3.2–4.4 ppm (2H × 0.95*n*, –NHC*H*_2_CO– for NAGAm units; 3H × 0.05*n*, –COOC*H*_3_ for NASMe units; 2H × 0.05*n*, –C*H*_2_OH for NASMe units; 1H × 0.05*n*, –COC*H*NH– for NASMe units).

### 4.5. Preparation of Hydrogel and Its Post-Crosslinking Reaction

The polymer solution was prepared by heating the P(NAGAm-*co*-NASMe) powder in water (~90 °C) using a microwave oven. Then, the aqueous solution was poured into silicone molds with different shapes, sandwiched between hydrophobically treated glass plates, and then cooled to 4 °C to prepare the hydrogels. In this study, 4 wt % hydrogels were used in all experiments. The post-crosslinking of the hydrogel by GA was conducted by immersing the gels in a GA aqueous solution for 1 h at 4 °C, which contained GA of 0, 1, 10, and 100 equivalents against NASMe units. Then, the resultant hydrogels were washed with pure water at 4 °C to remove any unreacted GA.

### 4.6. Shape Memory Experiments

The thermo-induced shape fixation and memory behaviors were characterized as follows. The straight-shaped hydrogels (θ_0_ = 180°) were first immersed in water at 25 °C for 1 h and then bent in half (deformation). Subsequently, the hydrogels were immersed in cold water at 4 °C for 1 h under an external force to fix their shapes. The bending angles of the temporarily shaped gel were measured after removing the external force (θ_1_). Finally, the hydrogels with temporary shapes were immersed in water at 25 °C to observe their shape recovery behaviors. The shape-fixing percentage *F* (%) was calculated using Equation (1). In addition, the shape recovery percentage *R* (%) was defined using Equation (2), where θ_2_ is the bending angle of the gel after recovery. Five independent tests (*N* = 5) were performed to evaluate the *F* and *R* values, and the average values were used.

Shape fixing percentage (*F*) (%) = [(θ_0_ − θ_1_)/θ_0_] × 100 (1)

Shape recovery percentage (*R*) (%) = (θ_2_/θ_0_) × 100 (2)

### 4.7. Swelling Behavior of Hydrogels

The swelling percentages (SP) of the gels were calculated as follows. The gel with a size of 1 mm × 10 mm × 10 mm was swelled for 24 h at 4 °C to reach swelling equilibrium. Then, the gel was immersed alternately in water at 25 and 4 °C for 1 h. The weight of gel (W_s_) at each temperature was measured after wiping the surface using absorbent paper. From the weight of the dried hydrogel, (W_d_), and W_s_, the SP was calculated using Equation (3).

SP (%) = [(W_s_ − W_d_)/W_d_] × 100 (3)

Four independent tests (*N* = 4) were performed to evaluate the SP values, and the average values were used.

### 4.8. Self-Healing Behavior of Hydrogels

The hydrogels were prepared by pouring the polymer solution (~90 °C) into a dumbbell-shaped silicone mold and cooling to 4 °C. The prepared hydrogels were cut into two pieces at the center using a razor blade. The cut pieces were placed back into the mold in tight contact and sandwiched between hydrophobically treated glass plates, then heated at 70 °C for 30 min and cooled at 4 °C for 10 min. Breaking energy (BE) was evaluated using a tensile test. The self-healing efficiency (HE), defined as the ratio of the breaking energies of the original (BE_o_) and self-healed gels (BE_h_), was calculated using the following Equation (4):

BE_h_/BE_o_ × 100 (4)

## Data Availability

Not applicable.

## Supplementary Materials

The following supporting information can be downloaded at: https://www.mdpi.com/article/10.3390/gels9100829/s1, Synthetic procedures of monomers. Supplementary figures (Figures S1–S4). ^1^H-NMR spectra of NAGAm, NASMe and P(NAGAm-*co*-NASMe): Temperature dependence of transmittance of P(NAGAm-*co*-NASMe) aqueous solution (1 wt %). Supplementary synthetic scheme (Scheme S1).
