# Supplementary material for: Mechanically Tunable Hydrogels with Self-Healing and Shape Memory Capabilities from Thermo-Responsive Amino Acid-Derived Vinyl Polymers
Source: Gels. 2023 Oct 19;9(10):829. doi: 10.3390/gels9100829 (PMC10606565; doi:10.3390/gels9100829)
Supplement: Supplementary file 1 [file gels-09-00829-s001.zip › gels-2666886-supplementary.pdf]

[*Supporting Information*]

Mechanically tunable hydrogels with self-healing and shape memory capabilities from thermo-responsive amino acid-derived vinyl polymers

Shin-nosuke Nishimura[1*], Dan Sato[1], Tomoyuki Koga[1*]

Synthesis of *N*-acryloyl glycinamide (NAGAm)

H-Gly-NH_2・HCl (6.00 g, 54.3 mmol) was dissolved in 2 M potassium carbonate aqueous solution (50 mL). To this solution, acryloyl chloride (6.60 mL, 81.7 mmol) was added slowly dropwise with stirring under an ice bath. The reaction was continued for 2 h at ambient temperature with stirring. After the reaction, 3 M HCl aqueous solution was added to adjust the pH 2 or lower. This protonated the carboxy groups of the byproduct acrylic acid, making it less soluble in water. The solution was then washed with diethyl ether (150 mL) to remove the acrylic acid. After performing this operation three times, the aqueous layer was collected and removed residual diethyl ether by standing for overnight at ambient condition. 3 M NaOH aqueous solution was added to the aqueous layer to adjust the pH to 7, and the resulting solution was lyophilized to give a crude solid. A methanol/ethanol mixture (v/v=1/4) was added to the crude and stirred for 30 min to extract the target product. The undissolved solid was removed by filtration. The resultant solution was concentrated under reduced pressure, cooled, and recrystallized to obtain a pure NAGAm as a white solid.

Synthesis of *N*-acryloyl serine methyl ester (NASMe)

H-Ser-OMe hydrochloride (4.97 g, 31.8 mmol) and DIPEA (11.1 mL, 63.2 mmol) were dissolved in DCM (200 mL) and cooled under an ice bath. Acryloyl chloride 2.56 mL (31.5 mmol) was diluted with DCM (15 mL) and added dropwise to the amino acid solution. After the addition, the reaction solution was stirred overnight at room temperature. The reaction mixture was washed five times with sat. NaCl aqueous solution (100 mL). The organic layer was collected and dried over anhydrous Na_2SO_4. The solution was concentrated *in vacuo* and then passed through a silica gel column using DCM/acetone/methanol (v/v/v=5/1/1) mixed solution as an eluent. The resultant solution was concentrated *in vacuo* and added a small amount of water. The aqueous solution was lyophilized to give a pure NASMe as a white solid.

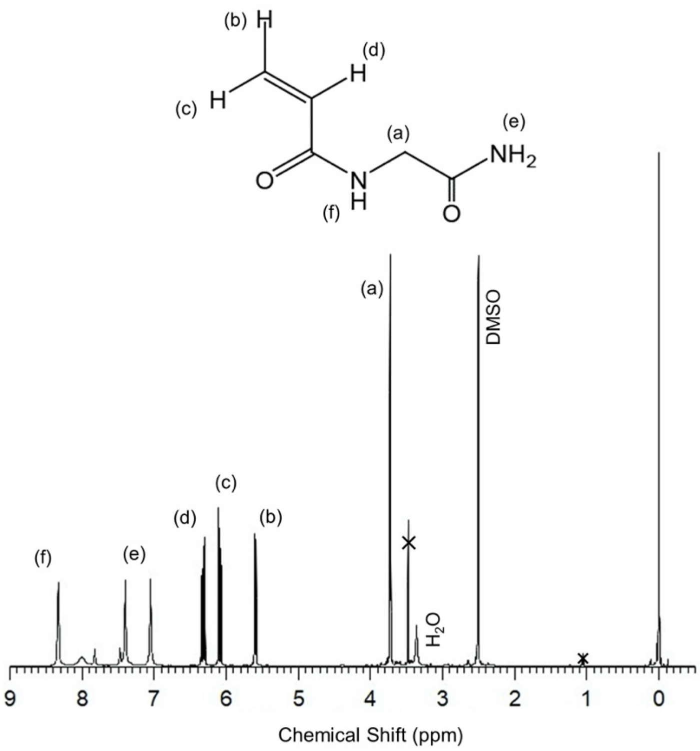

Figure S1. ^{1}H-NMR spectrum of *N*-acryloyl glycinamide (NAGAm) in DMSO-d_6 at 25°C (TMS standard).

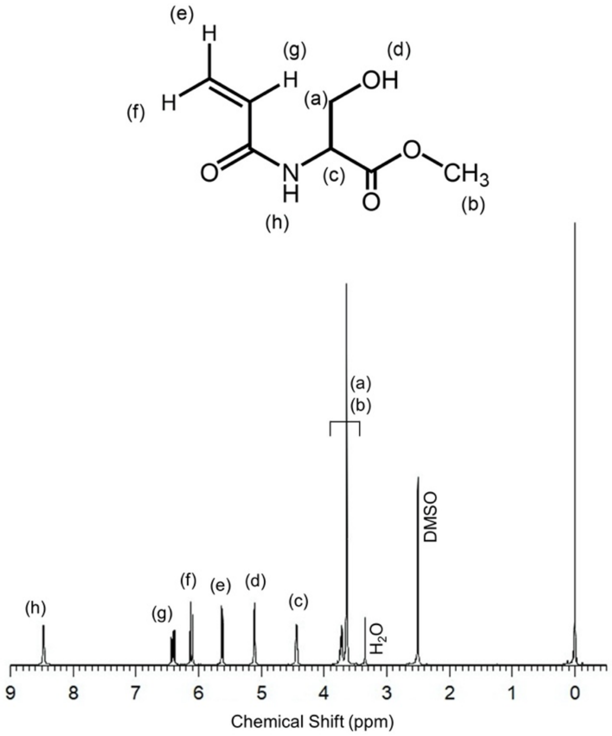

Figure S2. ^{1}H-NMR spectrum of *N*-acryloyl serine methyl ester (NASMe) in DMSO-d_6 at 25°C (TMS standard).

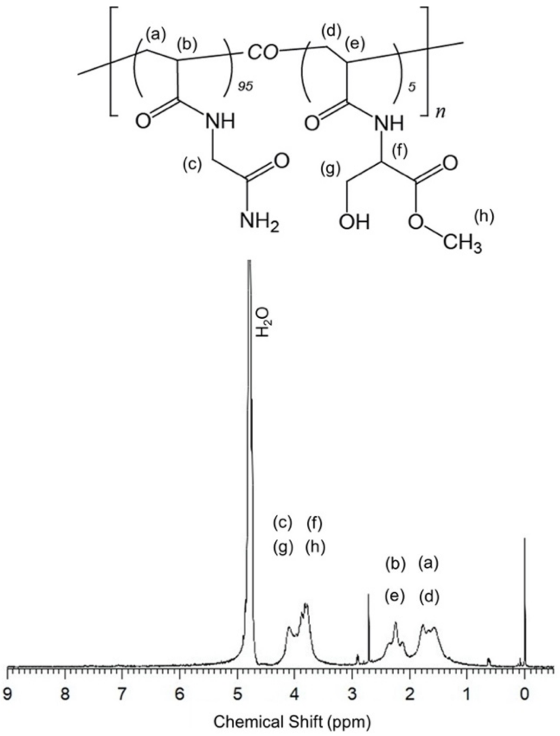

Figure S3. ^{1}H-NMR spectrum of poly(*N*-acryloyl glycinamide-*co*-*N*-acryloyl serine methyl ester), P(NAGAm-*co*-NASMe), in D_2O at 25°C (DSS standard).

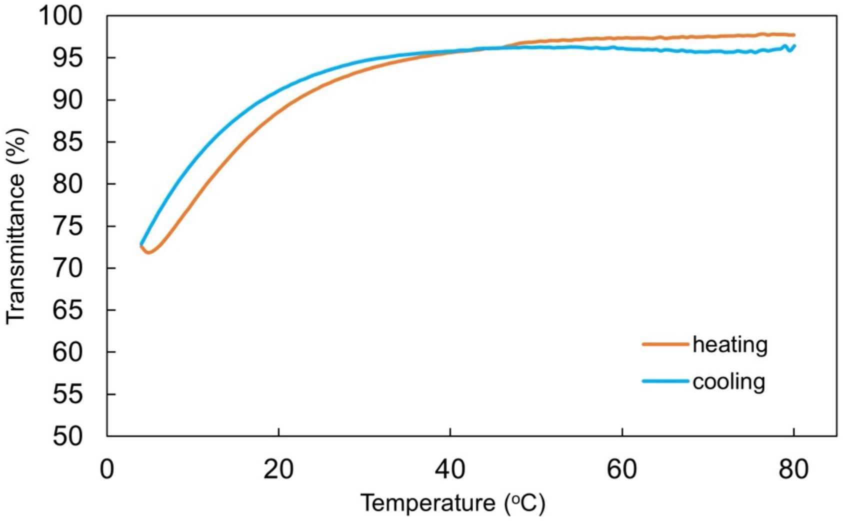

Figure S4. Temperature dependence of the transmittance of P(NAGAm-*co*-NASMe) aqueous solution (1 wt%) at 700 nm.

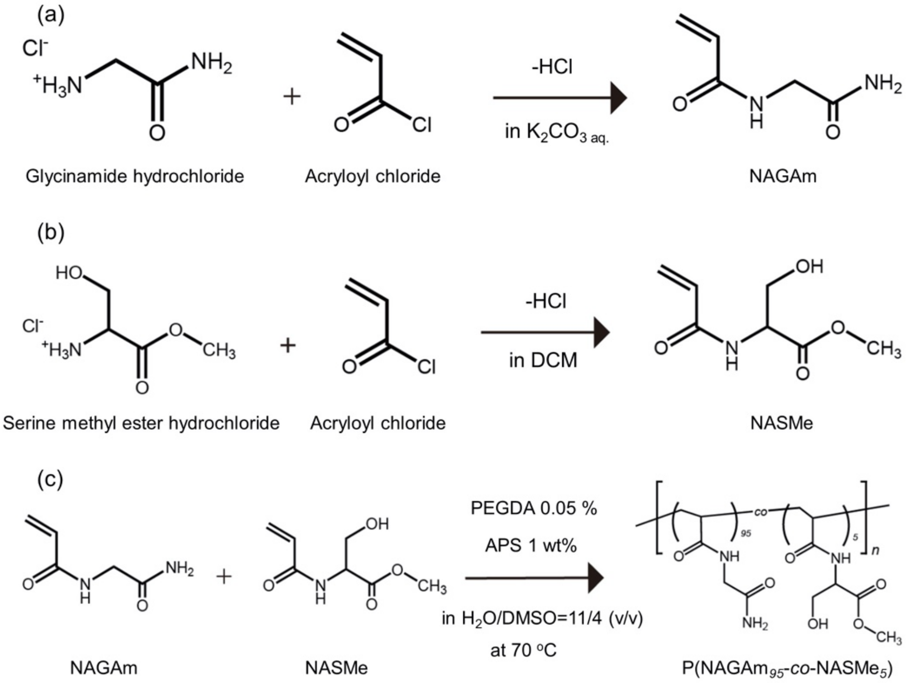

Scheme S1. Synthetic route of the P(NAGAm-*co*-NASMe).
